# Hope and challenges in the diagnosis and treatment of Wilms tumor: a single-center retrospective study in China

**DOI:** 10.3389/fped.2025.1527039

**Published:** 2025-04-14

**Authors:** Kongkong Cui, Peng Hong, Jie Lin, Zaihong Hu, Zhiqiang Gao, XiaoMao Tian, Tao Lin, Qinlin Shi, Guanghui Wei

**Affiliations:** ^1^ Department of Urology, Children’s Hospital of Chongqing Medical University, National Clinical Research Center for Child Health and Disorders, Ministry of Education Key Laboratory of Child Development and Disorders, Chongqing, China; ^2^ Chongqing Key Laboratory of Structural Birth Defect and Reconstruction, Chongqing, China

**Keywords:** Wilms tumor, prognosis, outcomes, abandonment, global review

## Abstract

**Background:**

Wilms tumor (WT), which represents about 90% of kidney tumors in children, is the most prevalent type of renal tumor among children. In developed countries, advancements in treatments such as chemotherapy and radiotherapy have led to high survival rates. However, developing countries face significant challenges, including late-stage diagnosis, metastasis at presentation, and high rates of treatment abandonment.

**Methods:**

This retrospective study included all patients diagnosed with WT at a tertiary hospital in Western China from 2007 to 2021. It involved the collection of sociodemographic and clinical details, including data on patients who abandoned treatment. Follow-up continued until July 2024.

**Results:**

This study consisted of 301 WT patients. Of the 259 who completed the treatment, the 5-year event-free survival (EFS) and overall survival (OS) rates were 77.9% and 81.2%. Of the 42 patients who abandoned treatment, 13 refused further care immediately after diagnosis and signed a refusal document, 16 discontinued treatments during preoperative neoadjuvant chemotherapy, and 13 failed to complete the prescribed chemotherapy or radiotherapy.

**Conclusion:**

Survival rates for WT patients at our institution approach those reported in developed countries. Challenges include late-stage diagnosis, metastasis at initial presentation, and treatment abandonment. To address these issues, implementing pediatric screening is critical for early detection and timely intervention, particularly for families vulnerable to abandoning treatment. For high-risk cases, oncologists need develop targeted strategies to enhance clinical outcomes.

## 1 Introduction

Wilms tumor (WT), which represents about 90% of kidney tumors in children, is the most prevalent type of renal tumor among children (1). Treatment usually consists of chemotherapy, surgery and, in some cases, radiotherapy. Two main treatment strategies are used globally. The approach practiced by the Children's Oncology Group (COG) in North America involves upfront surgery, whereas the European strategy, recommended by the International Society of Pediatric Oncology (SIOP), begins with preoperative chemotherapy. Extensive clinical trials have led to the development of integrated multi-modal therapies including surgery, chemotherapy and radiotherapy, with both strategies achieving similar long-term survival rates in high-income countries. Currently, most developed countries report 5-year survival rates exceeding 90% for early-stage disease and 70% for metastatic disease (2). However, data from developing countries indicate poorer outcomes, with most studies showing 5-year survival rates below 50% (3, 4). These differences may be due to increased incidence rates, delayed presentation and advanced disease at diagnosis (5). Additionally, treatment abandonment is a significant issue, defined as the failure to initiate or continue the prescribed treatment regimen after diagnosis or any interruption of more than 4 weeks (6). Although the critical impact of treatment abandonment on treatment failure is well recognized, the characteristics of patients who discontinue treatment and the factors influencing abandonment are poorly understood.

China, as one of the largest developing countries, reports a WT incidence of approximately 3.3 per million children. The incidence of WT among children is rising annually. However, in China, the capacity for effective treatment remains limited. Currently, only a few pediatric oncology centers report 5-year survival rates as high as 80% (7). Each center implements different treatment protocols based on its own experience. This study evaluated the characteristics and outcomes of WT patients treated at a tertiary care hospital in western China. The study also attempted to identify the characteristics of WT patients who abandoned treatment and the factors that influenced this decision.

## 2 Materials and methods

### 2.1 Treatment and follow-up

For patients with suspected WT, an experienced clinician will make the initial diagnosis by combining clinical performance and ultrasound, and then further clarification will be made by imaging tests such as CT and MRI. If the patient undergoes surgery or biopsy, the pathologic diagnosis prevails. Prior to initiating treatment, all patients undergo chest CT scans and abdominal contrast-enhanced CT scans. Neoadjuvant chemotherapy is selected as the initial treatment for cases involving an inferior vena cava tumor thrombus, invasion of adjacent organs (such as the spleen, pancreas, or colon), large tumor size (typically >12 cm in diameter) precluding complete resection, distant metastases, or bilateral WT. The chemotherapy regimen is administered according to the SIOP-WT 2001 protocol, utilizing a combination of two or three agents (vincristine and actinomycin D, with doxorubicin added if metastasis is present). Patients undergoing direct surgery were staged according to the COG system (8). The primary surgical approach for most patients is open nephrectomy via a transabdominal or thoracoabdominal incision. Laparoscopic nephrectomy may be considered as an alternative when the following criteria are met: (1) absence of extrarenal tumor infiltration, (2) no tumor thrombus in the renal vein or inferior vena cava, (3) tumor confinement within the ipsilateral spinal margin, and (4) minimal risk of intraoperative tumor rupture. Nephron-sparing surgery was prioritized in cases involving isolated kidneys, bilateral WT, horseshoe kidneys, or genetic syndromes such as Denys-Drash or Frasier syndromes. Because there is no radiotherapy equipment in our hospital, we had to recommend the patients who needed radiotherapy to the general hospital.

All WT patients require regular follow-up after surgery. According to our institution's practice, follow-up visits are scheduled once every 3 months during the first 3 years, once every 6 months from the fourth to the fifteenth year, and annually thereafter. Follow-up is conducted through outpatient examinations or telephone contact. Routine clinical examinations include physical examination, blood tests, and ultrasound. Follow-up was completed until the patient's death or until July 2024, whichever came first.

### 2.2 Study design

This study was approved by the Institutional Review Board (NO. 324 of 2024).

Between 2007 and 2021, Children's Hospital Affiliated to Chongqing Medical University (CQMU) enlisted children diagnosed with WT by an experienced pediatric oncologist. This identification was facilitated through enhanced CT scans and renal ultrasound assessments. We collected research data included pathology results, imaging data, and medical records. For follow-up, data were sourced from telephone interviews with families and outpatient records. The event-free survival (EFS) was defined as the time from admission to first disease recurrence, metastasis, death from any cause, or last follow-up. The overall survival (OS) was defined as the time from admission to death from any cause or last follow-up. Treatment abandonment was specifically defined as a disruption in treatment exceeding 4 weeks or failing to start or maintain the therapy post-diagnosis (6).

For tumor measurement, the volume was determined using the ellipsoid method from enhanced abdominal CT scans, calculated as length [cm] * width [cm] * depth [cm] * π/6 = volume [ml], and the largest tumor diameter was noted. To assess treatment efficacy, the Response Evaluation Criteria in Solid Tumors (RECIST) version 1.1 was utilized. This established metric categorizes tumor responses into four groups: complete response (CR), partial response (PR), stable disease (SD), and progressive disease (PD). CR indicates the total disappearance of all targeted lesions; PR denotes a reduction of at least 30% in total lesion diameters; PD is defined by at least a 20% increase in total lesion diameters compared to the smallest measured during the study; SD is identified when lesion sizes do not meet the reductions for PR or exceed (9).

In addition, we conducted a comprehensive global review of the literature on WT survival in various countries and summarized the relevant research findings. Furthermore, we performed a comparative analysis of the 5-year overall survival rates across different countries and regions. When multiple pertinent studies were available for a specific country or region, two experienced oncology research experts evaluated these studies based on several criteria, including sample size, data completeness, data reliability, study duration, and the initiating institutions. The most appropriate study was then selected for inclusion in the comparison.

### 2.3 Statistical analysis

Statistical analyses were conducted using R, version 4.2.3. Descriptive statistics were employed to assess demographic, socioeconomic, and clinical variables to identify differences between patients adhering to treatment regimens and those who abandoned them. Quantitative data were reported as medians (interquartile ranges). The Mann–Whitney *U* test was used to compare medians between groups. Categorical variables were presented as frequencies and percentages. Depending on the expected frequency counts, either Pearson's Chi-square test or Fisher's exact test was used to assess the differences. Multivariate logistic regression analysis was conducted for variables showing significant differences (*P* < 0.05) in the univariate analysis between the adherence and abandonment groups. The Kaplan–Meier method was employed to conduct survival analyses, and the Log-rank test was utilized to assess differences in survival rates. Statistical significance was established at a *P*-value of 0.05. Variables that demonstrated a *P*-value below 0.05 in the univariate analysis were further analyzed through multivariate Cox regression. Tumor stage was determined based on the COG protocol. To address issues of statistical instability due to limited sample sizes, early-stage patients (Stages I and II) were grouped into a low-stage category (*n* = 125). Similarly, later-stage patients (Stages III, IV, and V) were classified into a high-stage category (*n* = 134).

## 3 Results

### 3.1 Sociodemographic and clinical characteristics

Between 2007 and 2021, 339 patients with renal tumors were identified at CQMU (Supplementary Figure 1). Following exclusion of 38 cases with pathologically confirmed non-WT diagnoses, the remaining 301 WT patients were clinically evaluated by senior oncologists and confirmed through standardized imaging protocols. Sociodemographic and clinical details, including data on WT patients who abandoned treatment, are presented in Table 1. The most common symptoms included an abdominal mass in 200 patients (66.5%), hematuria in 44 (14.6%), abdominal pain in 26 (8.6%), and various other symptoms in 31 (10.3%). Additional clinical presentations included abdominal distention (*n* = 8), fever (*n* = 9), incidental trauma-related findings (*n* = 3), weight loss (*n* = 2), and incidental physical examination findings (*n* = 5). Prenatal detection of fetal tumors via ultrasound was documented in four cases. The median age at diagnosis was 27 months (IQR: 14–46 months). Of these children, 143 (47.5%) were male and 158 (52.5%) were female. The average distance of residence from CQMU was 213 km (IQR: 93–309 km). Among the 259 WT patients who adhered to treatment, there were 13 bilateral cases. Among these, 4 underwent unilateral nephrectomy and Nephron-Sparing Surgery on the other side, while 9 received bilateral Nephron-Sparing Surgery. In the remaining 246 unilateral WT patients, 23 underwent Nephron-Sparing Surgery, and 223 patients received unilateral radical nephrectomy. Notably, only three of these cases were performed with laparoscopic assistance.

**Table 1:** Clinical and sociodemographic profile of WT patients admitted between 2007 and 2021.

Characteristics	Total (*n* = 301)	Adhered to treatment (*n* = 259)	Abandoned treatment (*n* = 42)	*P* value
Age (months)	27 (14–46)	27 (15–46)	20 (9–47)	0.268
Sex				0.325
Male	143 (47.5%)	126 (48.7%)	17 (40.5%)	
Female	158 (52.5%)	133 (51.4%)	25 (59.5%)	
Tumor Stage				N/A
Ⅰ	N/A	47 (18.2%)	N/A	
Ⅱ	N/A	78 (30.1%)	N/A	
Ⅲ	N/A	88 (34.0%)	N/A	
Ⅳ	N/A	33 (12.7%)	N/A	
Ⅴ	N/A	13 (5.0%)	N/A	
Type				N/A
FH	N/A	209 (80.7%)	N/A	
uFH	N/A	50 (19.3%)	N/A	
Area of residence				<0.001
Rural	180 (59.8%)	145 (56.0%)	35 (83.3%)	
Urban	121 (40.2%)	114 (44.0%)	7 (16.7%)	
Medical insurance				<0.001
Yes	143 (47.5%)	134 (51.7%)	9 (21.4%)	
No	158 (52.5%)	125 (48.3%)	33 (78.6%)	
Duration^a^	8 (4–30)	7 (4–30)	24 (7–58)	<0.001
Tumor site				0.623
Left	149 (49.5%)	129 (49.8%)	20 (47.6%)	
Right	139 (46.2%)	120 (46.3%)	19 (45.2%)	
Bilateral	13 (4.3%)	10 (3.9%)	3 (7.1%)	
Symptoms				0.020
Abdominal Mass	200 (66.5%)	174 (67.2%)	26 (61.9%)	
Hematuria	44 (14.6%)	36 (13.9%)	8 (19.1%)	
Abdominal Pain	26 (8.6%)	26 (10.0%)	0 (0%)	
Others	31 (10.3%)	23 (8.9%)	8 (19.1%)	
Neoadjuvant Chemotherapy				0.017
Yes	83 (25.8%)	65 (25.1%)	18 (42.9%)	
No	218 (72.4%)	194 (74.9%)	24 (57.1%)	
Metastasis				<0.001
Yes	38 (12.6%)	17 (6.6%)	21 (50.0%)	
No	263 (87.4%)	242 (93.4%)	21 (50.0%)	
Tumor thrombus inferior vena cava/renal vein				0.727
Yes	38 (12.6%)	32 (12.4%)	6 (14.3%)	
No	263 (87.4%)	227 (87.6%)	36 (85.7%)	
Distance^b^ (km)	213 (93–309)	216 (100–310)	190 (81–237)	0.295

^a^ This refers to the number of days elapsed from the initial detection of symptoms to the patient's first visit to CQMU.

^b^ Driving directions with Google Maps from address provided by family to CQMU.

### 3.2 Response to neoadjuvant chemotherapy

In this cohort study, 83 patients received neoadjuvant chemotherapy. Fourteen patients were excluded due to incomplete pre- or post-chemotherapy imaging. Tumor size was assessed using contrast-enhanced CT scans before and after treatment in the remaining 69 patients, all of whom had complete clinical records available for analysis. Among them, 26 patients (38%) achieved a PR, 7 patients (10%) experienced PD, and 36 patients (52%) exhibited SD. Initially, the average maximum tumor diameter was 14 cm, which decreased to 11 cm after treatment, representing a 21% reduction. Similarly, the average tumor volume decreased from 813 to 552 ml, a 32% reduction.

### 3.3 Received treatment

In this study, 259 patients adhered to the treatment plan. Among these, 51.7% presented with high-stage disease, and 258 underwent either radical nephrectomy or nephron-sparing surgery. One child, who had previously undergone a unilateral nephrectomy due to a renal laceration, developed a retroperitoneal mass 7 months postoperatively, necessitating a subsequent retroperitoneal tumor resection. During these surgeries, 59 cases were found to have either an incomplete tumor capsule or tumor rupture. Furthermore, tumor invasion of the duodenum was observed in one patient, while another had tumor invasion in both the duodenum and the descending colon. Among the adherent patients, 65 received preoperative chemotherapy. Excluding those who delayed chemotherapy or altered their chemotherapy drugs due to personal health issues, the remainder completed their treatment as planned.

### 3.4 Abandoned treatment

**Figure 1:**
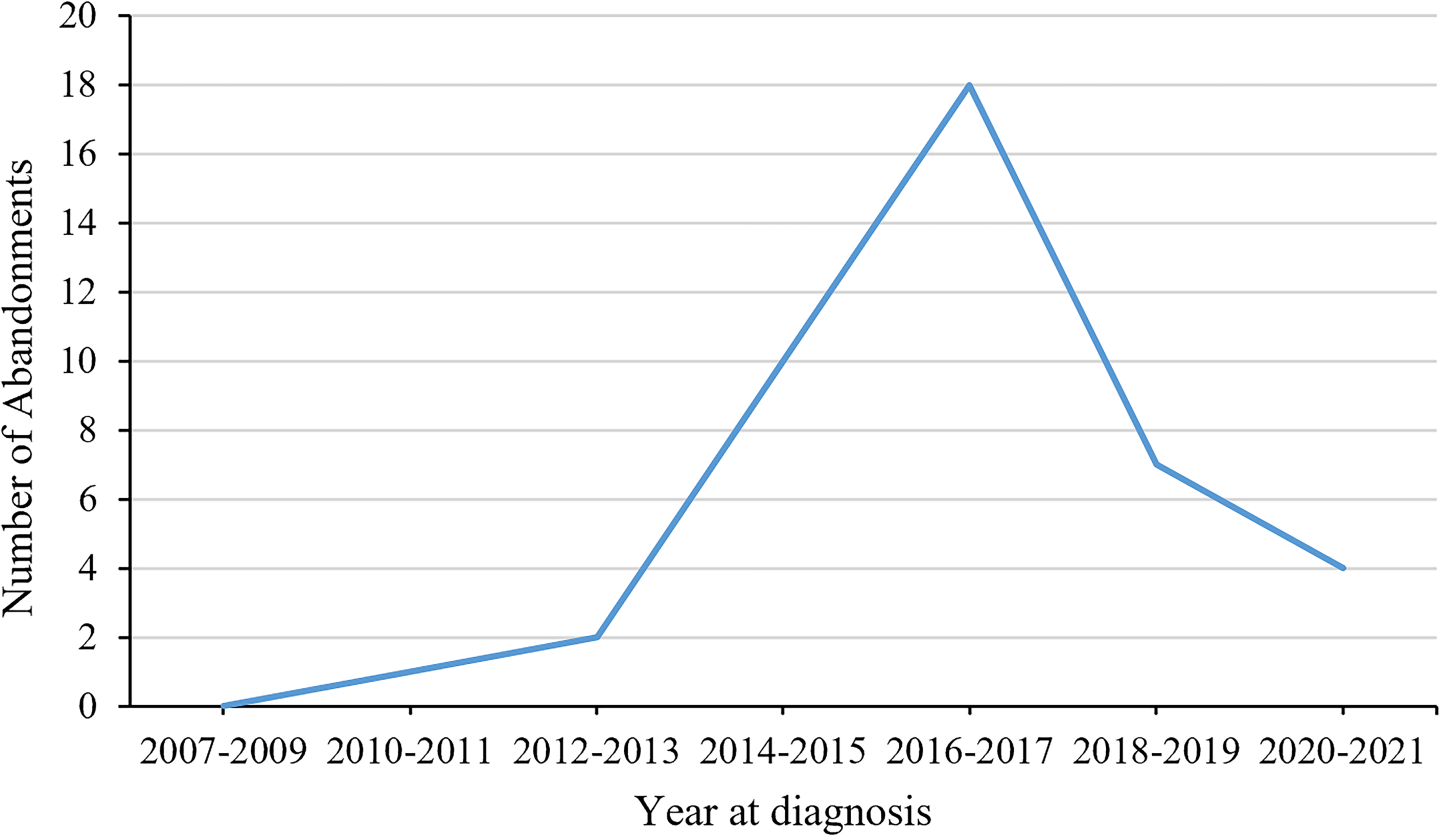
Annual number of children abandoning treatment.

In the cohort of 42 patients who abandoned their treatment, 13 chose to cease further medical intervention post-diagnosis, as documented by their signing of a refusal form. Additionally, 16 patients ceased participation during the preoperative neoadjuvant chemotherapy stage, and 13 were classified as having abandoned treatment due to their failure to complete the designated chemotherapy or radiotherapy regimen. The number of patients who abandoned treatment each year is shown in Figure 1. Univariate analysis indicated significant correlations between treatment abandonment and factors such as area of residence, medical insurance status, duration of symptoms before initial admission, neoadjuvant chemotherapy, and metastasis. Given the wide and uneven distribution of symptoms, which can introduce instability and bias into regression models, these were not included in the multifactorial analysis. Multivariate regression analysis revealed that patients living in rural areas were more likely to abandon treatment compared to those in urban areas (OR 3.60, 95% CI 1.28–10.13, *P* = 0.015); those without medical insurance were more likely to abandon treatment compared to those with insurance (OR 5.28, 95% CI 1.96–14.17, *P* < 0.001); each additional day from symptom onset to initial consultation increased the likelihood of treatment abandonment by 1% (OR 1.01, 95% CI 1.01–1.02, *P* < 0.001); and patients presenting with metastatic disease at consultation were more likely to abandon treatment (OR 18.54, 95% CI 7.31–47.02, *P* < 0.001). For details, see Supplementary Table 1.

### 3.5 Treatment outcomes

**Figure 2:**
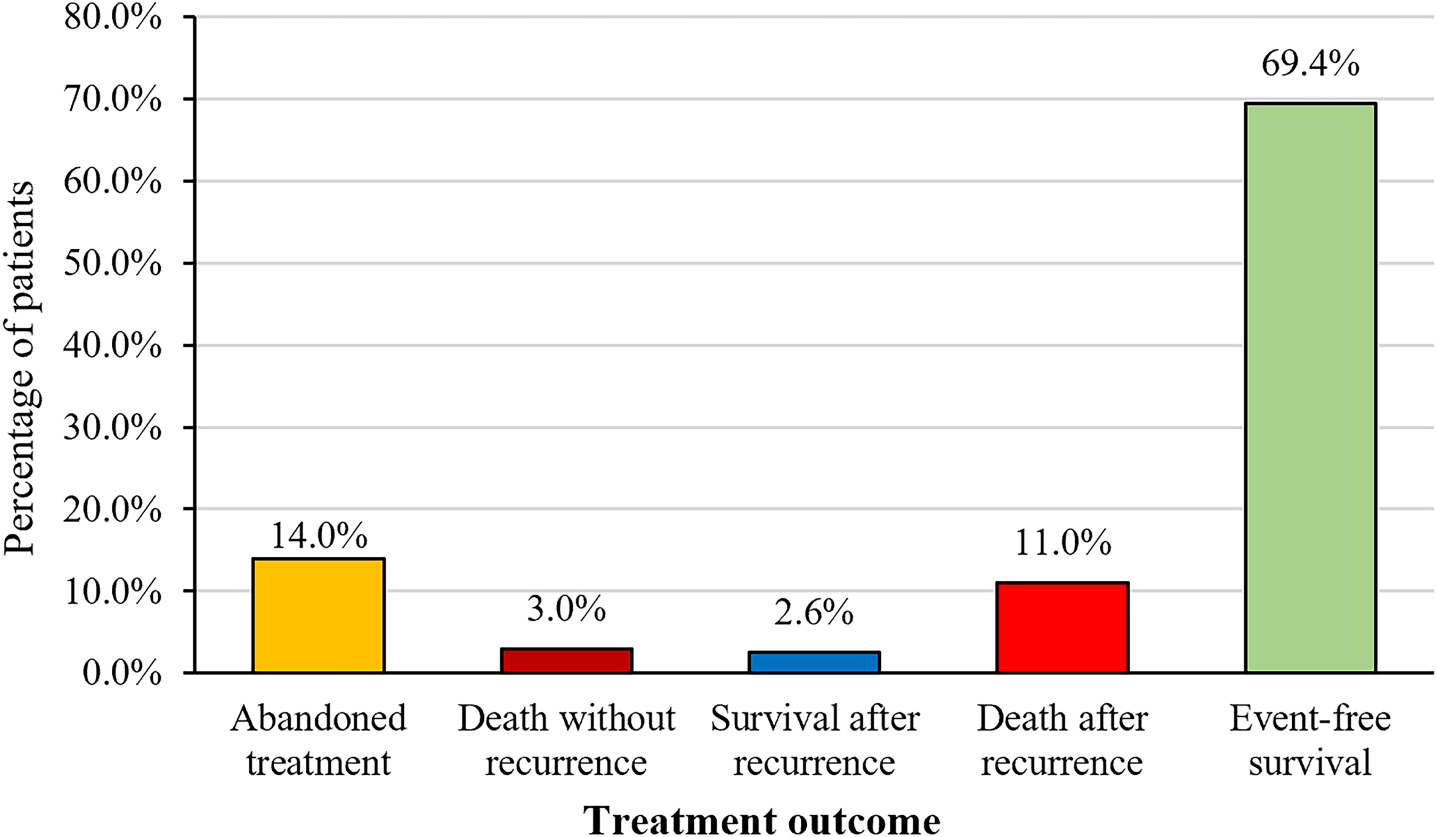
Treatment outcome of patients with WT (*n* = 301).

**Figure 3:**
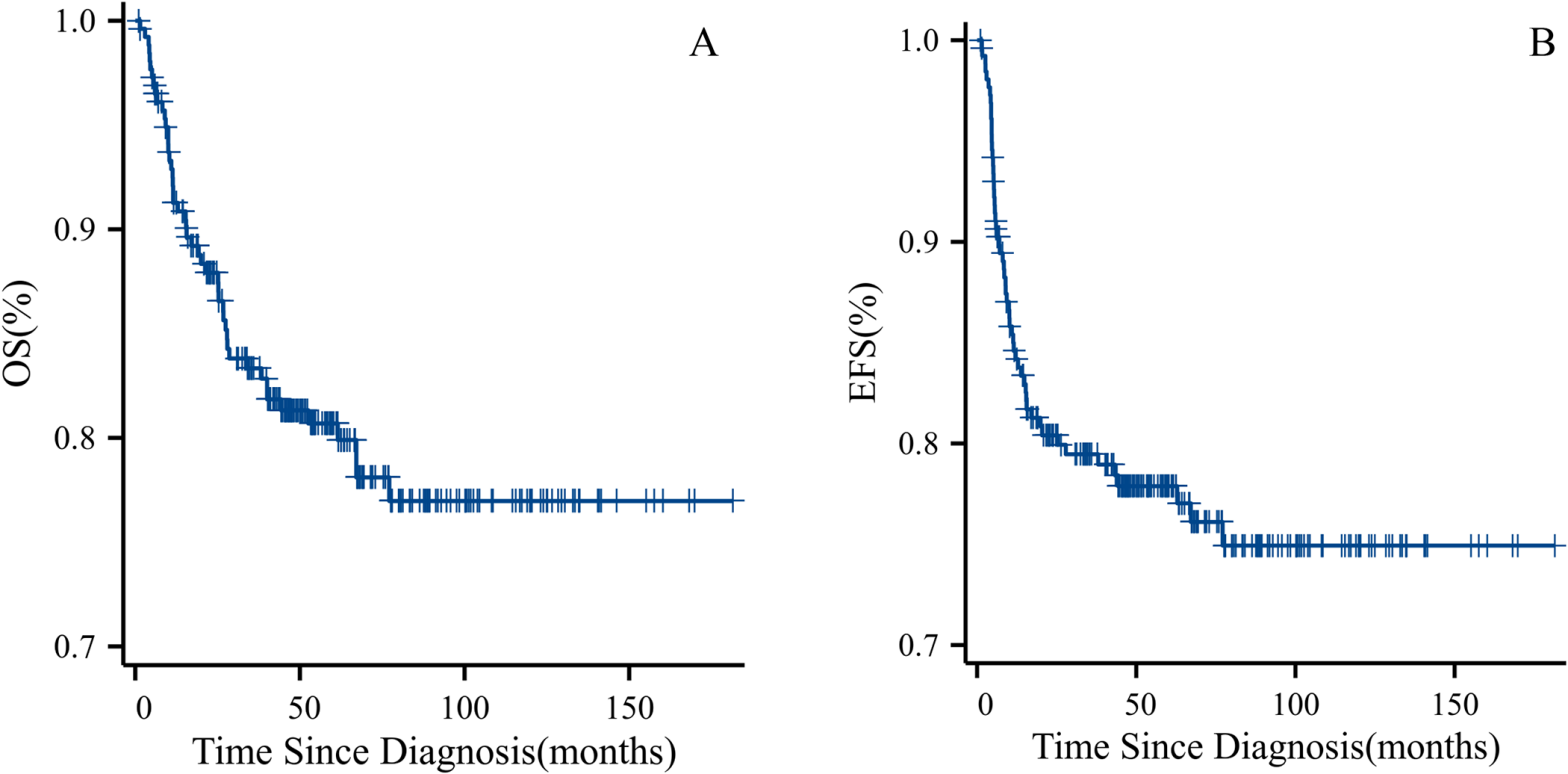
**(A)** OS for all patients who adhered to treatment. **(B)** EFS for all patients who adhered to treatment.

**Figure 4:**
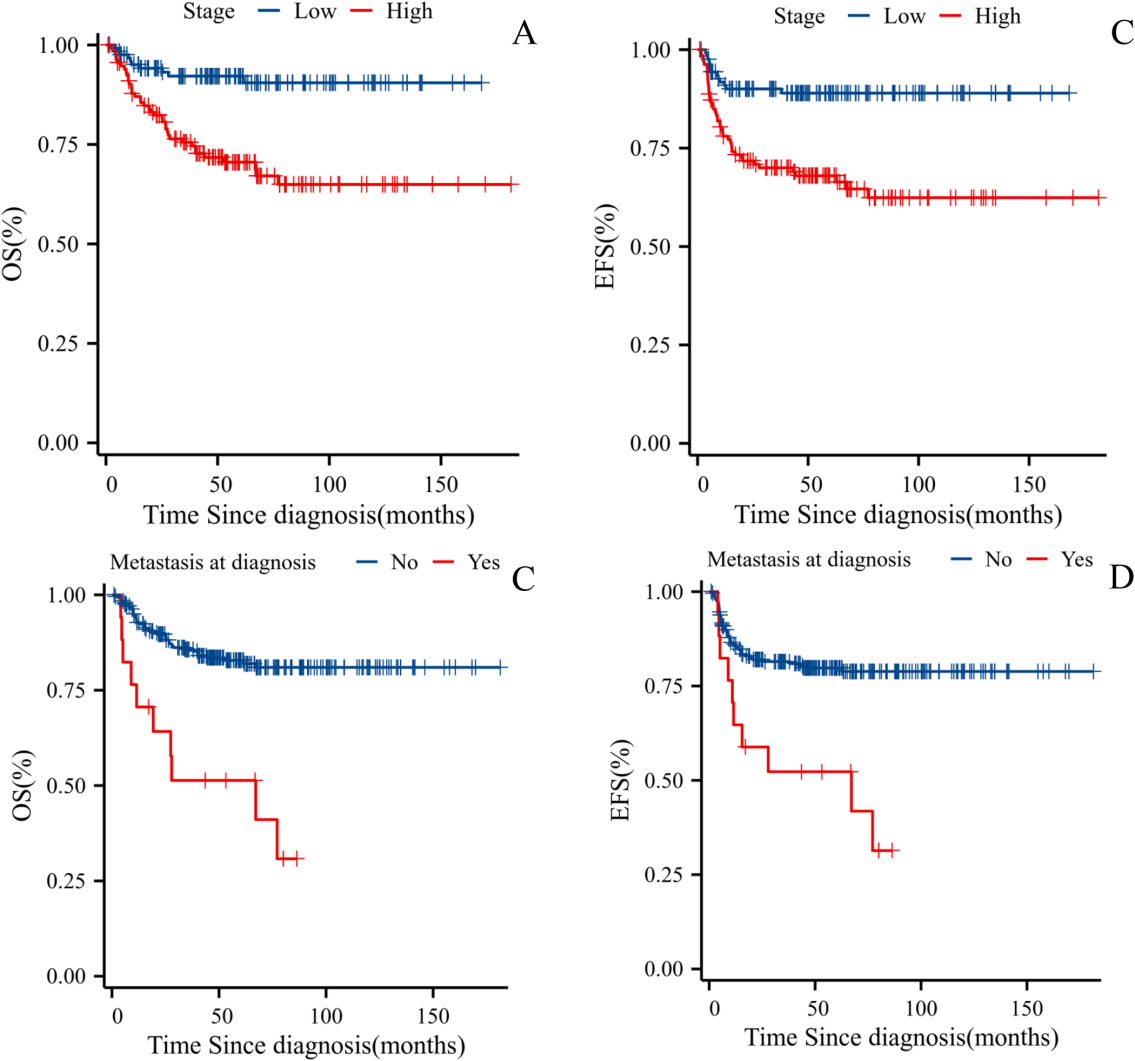
**(A)** OS for WT patients based on tumor stage. **(B)** EFS for WT Patients Based on Tumor Stage. **(C)** OS for WT Patients Based on Presence of Metastasis at Diagnosis. **(D)** EFS for WT Patients Based on Presence of Metastasis at Diagnosis.

Among the 301 patients, 42 opted to abandon treatment, while 259 received complete treatment (see Figure 2 for the Treatment Outcome of Patients with WT, *n* = 301). Our study focuses on these 259 patients who underwent complete treatment, with follow-up continuing until July 2024. The median follow-up duration was 57 months (range: 1–181 months). Seven patients died due to treatment-related causes (three from sepsis, one from respiratory failure, and three from severe electrolyte imbalances), 33 patients died from progressive disease, and 2 died at home due to unknown causes. The 5-year OS rate among the 259 patients was 81.2%, and the 5-year EFS rate was 77.9% (see Figure 3A,B). A total of 41 patients experienced relapse, resulting in 33 deaths. The locations for relapses were the tumor bed (*n* = 22), lungs (*n* = 20), liver (*n* = 9), mediastinal region (*n* = 4), pelvic area (*n* = 5), and intracranial regions (*n* = 2). A significant number of these patients had relapses at multiple sites, with the median time to relapse being 7 months (range: 2–63 months). We included factors with a *P*-value <0.05 from univariate analysis in the multivariate Cox regression analysis. The results indicated that high stage (*P* < 0.001, HR 2.9, 95% CI 1.56–5.59), metastasis (*P* = 0.048, HR 2.0, 95% CI 1.01–4.14), and the duration of symptoms before first admission (*P* = 0.012, HR 1.01, 95% CI 1.00–1.01) were significant adverse prognostic factors for EFS. High stage (*P* = 0.002, HR 3.1, 95% CI 1.52–6.42) and metastasis (*P* = 0.014, HR 2.52, 95% CI 1.21–5.25) were significant adverse prognostic factors for OS. The 5-year OS and EFS rates for the low-stage group were 92.2% and 89.0%, respectively, compared to 71.4% and 68.0% for the high-stage group. OS significantly differed between the low-stage and high-stage groups (*P* < 0.001), as did EFS (*P* < 0.001) (see Figure 4A,B). For the group with metastasis at diagnosis, the 5-year OS rate was 51.3%, and the EFS rate was 52.3%, compared to an OS rate of 83.3% and an EFS rate of 79.8% for the group without metastasis. OS and EFS significantly differed between the metastasis and non-metastasis groups (*P* < 0.001) (see Figure 4C,D).

### 3.6 Global review

We reviewed 46 studies from 44 countries or regions globally that investigated survival rates for WT. Among these, 12 studies identified histological subtype as the primary factor influencing the prognosis of WT patients, and 15 studies highlighted staging as a key prognostic factor, aligning with our findings. Our findings are consistent with the observation that tumor metastasis plays a significant role in determining prognosis, as highlighted in six studies. Moreover, tumor volume has been identified as a critical prognostic factor across six studies. In addition, three studies have proposed that female gender may be linked to poorer prognosis. Severe acute malnutrition was noted as a predictor of adverse outcomes in two studies, while another two studies identified age at the time of diagnosis as a significant prognostic indicator. Individual studies have also established connections between vena caval tumor involvement and poor prognosis, tumor rupture and unfavorable outcomes, and lymph node involvement as a key predictor of adverse prognosis.

**Figure 5:**
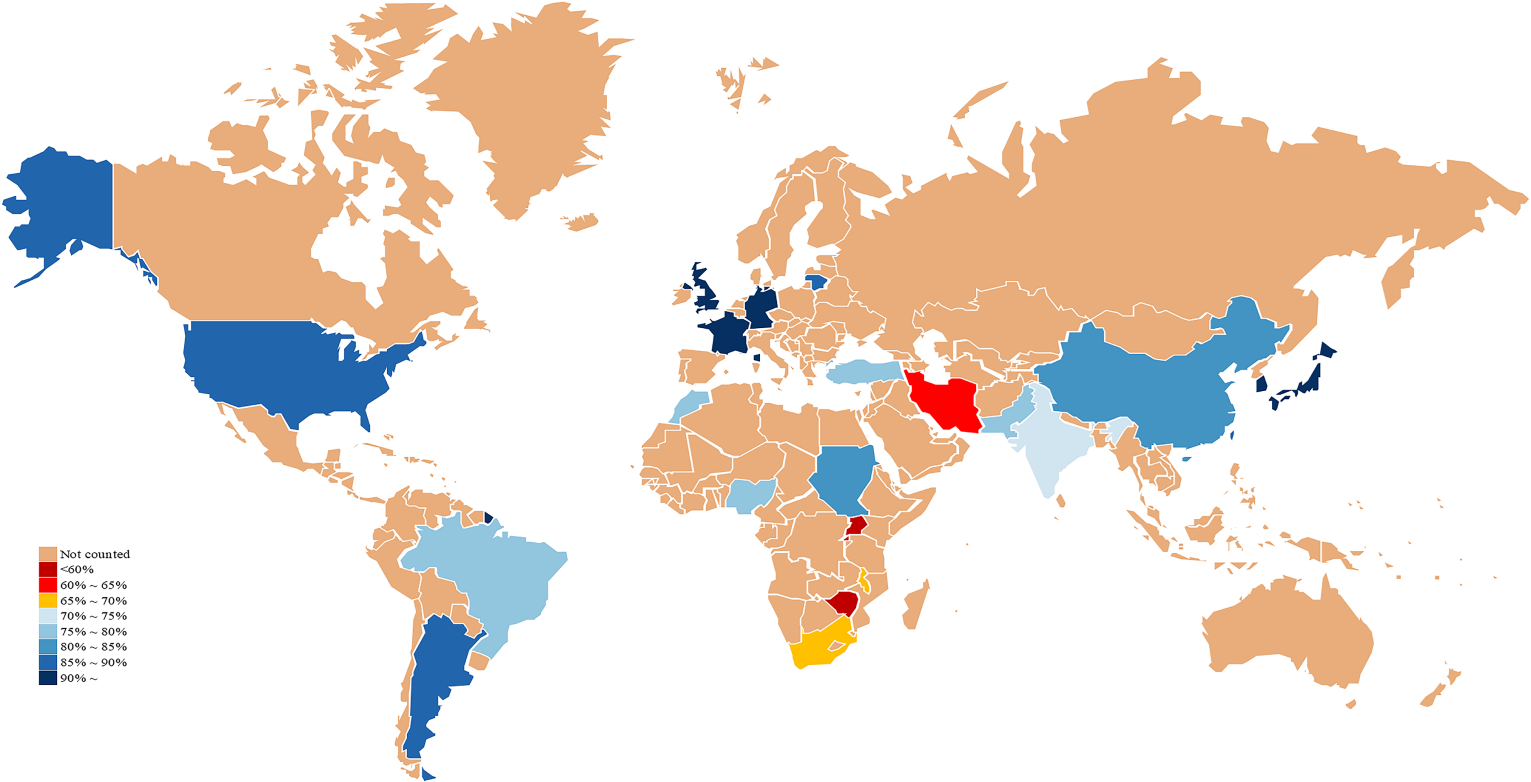
Global distribution of 5-year OS for WT by country. Map lines delineate study areas and do not necessarily depict recognized national boundaries.

In the analysis of the global WT 5-year OS, high-income countries such as France (98%), South Korea (97.2%), Germany (95%), the United Kingdom (91.4%), Japan (92.1%), and the United States (87%) generally exhibit high survival rates (above 85%). In contrast, low-income countries like Uganda (7.9%), Zimbabwe (33.2%), and Rwanda (48.3%) show significantly lower survival rates. Regionally, Europe, including France (98%), Germany (95%), Lithuania (86.4%), and the United Kingdom (91.4%), generally demonstrates higher survival rates. In Asia, there is considerable variation, with Japan having the highest rate (92.1%) and Iran the lowest (62%). In Africa, except for Morocco, which performs relatively well (79%), survival rates are generally low, with Uganda being the lowest at just 7.9%. The Americas also display some variation, with the United States (87%) showing a better survival rate compared to Brazil (75%). Figure 5 illustrates the global distribution of 5-year OS for WT by country. Involving China, five studies cover four regions: Shanghai, Yunnan, Hong Kong, and Taiwan. The highest survival rate is observed in Hong Kong (94%, 5-year), while the lowest is in Shanghai (81%, 4-year) (see Supplementary Table 2).

## 4 Discussion

In our study, WT represented 89% of all renal tumors in children, which is closely to the results of rates reported in prior literature (1). In this study, 97% of patients presented with symptoms such as abdominal mass, hematuria, or abdominal pain, while only 9 cases were asymptomatic and detected during routine examinations. At presentation, 12.6% of patients had metastatic disease, which contrasts with findings from high-income countries where most tumors are asymptomatic and detected through surveillance (10, 11). Early detection and timely medical consultations are critical for improving outcomes in these children. In our series of 69 WT patients received preoperative neo-adjuvant chemotherapy. The average tumor volume decreased from 813 to 553 ml with neo-adjuvant chemotherapy, resulting in a mean tumor size reduction of 32%. This response is lower than that reported in the SIOP-9 study, where the tumor size was reduced by 50% (12). Although the reduction in tumor volume in our study is lower than in other studies, most patients achieved the intended outcome with neo-adjuvant chemotherapy, which improved the rate of complete surgical resection and reduced the risk of tumor rupture. Neo-adjuvant chemotherapy also helped assess tumor sensitivity to chemotherapy, providing guidance for postoperative treatment (13). We achieved favorable outcomes, with a 5-year overall survival rate of 81.2% and a 5-year event-free survival rate of 77.9%. These results are comparable to those reported in developed countries (14).

Despite the generally high survival rates of WT, especially in patients with favorable histology or low-stage tumors, treatment abandonment remains a significant and preventable cause of mortality, contributing to survival disparities in developing countries (15). This study is the first to analyse treatment abandonment in Chinese WT patients and included 42 cases from 2007 to 2021. The most frequent stage for treatment abandonment occurred during neoadjuvant chemotherapy (38%), consistent with findings from Nanteza et al. (2024) (15). Several factors associated with treatment abandonment were identified, including rural residence, lack of medical insurance, extended duration from symptom onset to initial consultation, neoadjuvant chemotherapy, and metastasis. The impact of rural residence and lack of medical insurance, which often leads to treatment abandonment, was also highlighted in a large-scale study on childhood acute lymphoblastic leukemia in China (16). One study on treatment abandonment in pediatric acute lymphoblastic leukemia, the implementation of a government healthcare program decreased the abandonment rate from 50% to 20%, significantly reducing treatment abandonment cases (17). Strengthening medical assistance programs and providing direct financial support to families who cannot afford treatment could alleviate the phenomenon of abandonment due to economic reasons.

Based on a review of 46 studies involving 44 countries or regions, we found that survival rates are generally higher in developed countries such as those in Europe and North America, while lower survival rates are observed in African countries (2–5, 14, 18–59). We were particularly shocked to discover that Uganda has a 5-year OS of only 7.9% (3). Many countries have established pediatric oncology research organizations, such as JWiTS in Japan and KPHOG in South Korea, which typically adopt treatment protocols similar to those of SIOP or COG (14, 33). Our literature review identified advanced tumor stages, unfavorable histological subtypes, and metastasis as the most frequently reported risk factors for poor prognosis. We recommend that pediatric oncologists focus more on treating patients with these risk factors.

In the treatment of WT, although open surgery is still the standard approach, the use of minimally invasive surgery, especially robotic techniques, has gradually increased in recent years. In a previous study on adult WT, robotic radical nephrectomy after neoadjuvant chemotherapy achieved favorable treatment results. In the future this will also make one of the directions of development for pediatric WT (60).

In recent years, numerous biomarkers with significant prognostic implications for WT have been identified. Among these, particularly loss of heterozygosity (LOH) at 1p and 16q has demonstrated robust prognostic value. The NWTS-5 trial revealed that patients with 1p or 16q LOH exhibited significantly higher relapse and mortality rates, independent of tumor stage or histology (61). These findings have led to the integration of 1p/16q LOH into risk stratification within COG protocols. In addition, molecular alterations such as TP53 mutations are strongly associated with unfavorable histological subtypes, including diffuse anaplastic histology, as evidenced by multiple studies (62). Similarly, the SIOP-WT2001 trial highlighted MYCN gain as an independent prognostic marker linked to diffuse mesenchymal histology and poorer recurrence-free and overall survival outcomes (63). Emerging biomarkers, including Prohibitin (PHB), SIX1/2 mutations, and microRNA dysregulation, have also shown promise in refining risk prediction and therapeutic targeting (64–66). To advance precision oncology in WT, future research should prioritize the validation of these novel biomarkers while elucidating their mechanistic roles in tumorigenesis and progression. Such efforts will enhance risk-adapted therapies and ultimately improve clinical outcomes for WT patients.

## 5 Conclusions

Although survival rates for WT are generally favorable, significant challenges persist, especially in developing countries where advanced stages or metastasis are commonly observed at diagnosis. Early detection through routine pediatric screenings is essential. Addressing socioeconomic barriers to treatment, such as improving financial support, expanding insurance coverage, and enhancing education, can reduce treatment abandonment and improve survival outcomes. Our findings also underscore the importance of individualized treatment approaches, with a focus on optimizing therapies for patients with high-risk factors while seeking less harmful treatment options for those without such factors.

## Data Availability

The raw data supporting the conclusions of this article will be made available by the authors, without undue reservation.
