# Supplementary material for: Hope and challenges in the diagnosis and treatment of Wilms tumor: a single-center retrospective study in China
Source: Front Pediatr. 2025 Apr 14;13:1527039. doi: 10.3389/fped.2025.1527039 (PMC12034702; doi:10.3389/fped.2025.1527039)
Supplement: Supplementary file 1 [file Datasheet1.pdf]

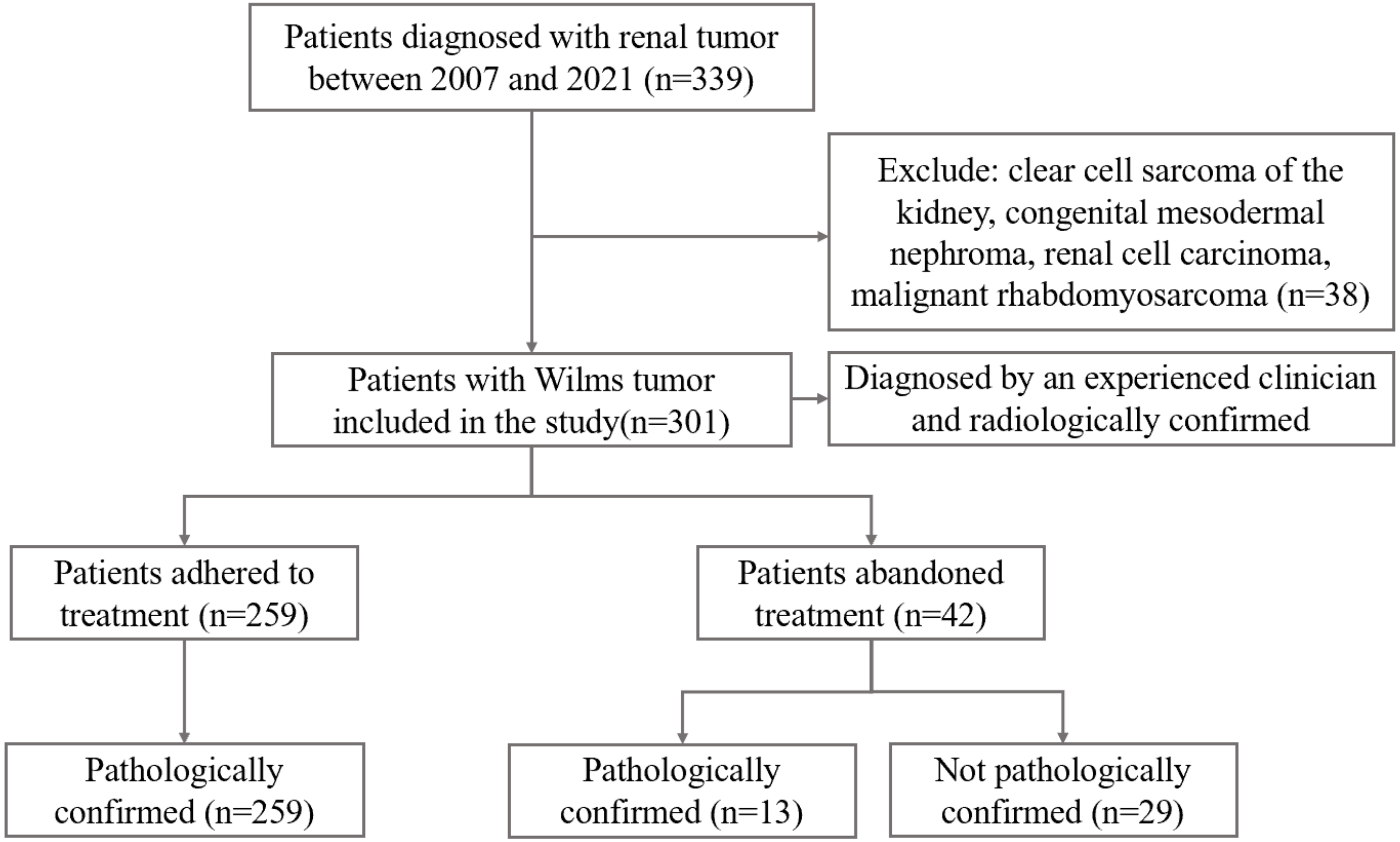

Supplementary figure1 Patients with Wilms tumor included in the study

Surgical procedure

Surgery is usually performed through a transabdominal or thoracoabdominal incision, which provides full visibility of the kidney. The incision should also be large enough to prevent rupturing the tumor through compression. Preoperative or intraoperative biopsies are avoided because they can increase the risk of tumor spread or metastasis. For resectable masses, biopsies are not performed. To minimize hematogenous tumor spread, the renal hilum is addressed first, whenever possible. If surgical difficulty arises, the mass may be freed before ligating the renal hilum. The goal is to remove the kidney and ureter while avoiding unnecessary extensive or destructive surgery. If the tumor is connected to neighboring organs, only a limited number of adjacent structures should be removed if it ensures complete tumor resection. In cases where more extensive surgery is not feasible, only a biopsy and chemotherapy may be performed prior to attempting surgery, and the ureters should be ligated at a low level whenever possible. When preoperative imaging confirms that the contralateral kidney is normal, no intraoperative exploration of that kidney is needed. Short tumor thrombi in the renal vein can be removed alongside the renal vein. For vena cava thrombi below the hepatic vein, the distal and proximal vena cava and contralateral renal vein are temporarily blocked, and a vessel wall incision allows for tumor thrombus removal. If the thrombi extend above the hepatic vein, extracorporeal circulation is required. Extensive thrombi infiltration into the vena cava wall necessitates weighing the risks and benefits, with radiotherapy as a potential alternative. If negative surgical margins from the tumor to the adrenal gland are clear, the adrenal gland is preserved. Otherwise, it is removed. Intraoperative exploration of the renal hilum, para-abdominal aorta, and contralateral perirenal hilum lymph nodes is mandatory for intraoperative lymph node biopsy, and the number of lymph node biopsies should be $\geqslant 7$. Titanium clips are used to mark the suspected residual tumor margins. Surgical exploration should progress methodically from a distant area to the target site to prevent tumor cell dissemination to other regions.

Supplementary table 1 Multivariate analysis of clinical characteristics associated with treatment abandonment

Characteristics	Adhered to treatment (n=259)	Abandoned treatment (n=42)	OR (95%CI)	*p* value
Area of residence				0.015
Urban	114(44.0%)	7(16.7%)		
Rural	145(56.0%)	35(83.3%)	3.60(1.28-10.13)	
Medical insurance				<0.001
Yes	134(51.7%)	9(21.4%)		
No	125(48.3%)	33(78.6%)	5.28 (1.96-14.17)	
Duration of symptoms before first admission[a]	7(4-30)	24(7-58)	1.01(1.01-1.02)	<0.001
Neoadjuvant Chemotherapy				0.305
No	194(74.9%)	24(57.1%)		
Yes	65(25.1%)	18(42.9%)	1.55(0.67-3.61)	
Metastasis				<0.001
No	242(93.4%)	21(50.0%)		
Yes	17(6.6%)	21(50.0%)	18.54(7.31-47.02)	

Supplementary table 2 Studies on WT in various countries around the world

No	Researcher	Year	Country /Region	Follow-up time (years)	Sample Size	Treatment Protocol	EFS%	OS%	Prognostic Factors
1 [b]	Yildiz	2000	Turkey	NA	106	NWTS-3, NWTS-4	72.4 (5-year)	76.6 (5-year)	NA
2	Reinhard	2004	Germany	NA	519	SIOP-93-01	91 (5-year)	NA	Metastasis, tumor volume, histology
3[b]	Hung	2004	China (Taiwan)	7.4 (0.15-10.68)	98	NA	78.4 (5-year)	88.6 (5-year)	Sex
4[b]	Madani	2005	Morocco	5.83	86	SIOP-9	77.4 (5-year)	79 (5-year)	NA
5[b]	Cotton	2009	America	NA	6185	NA	NA	87 (5-year)	NA
6	Akyüz	2010	Turkey	5.6 (0.50-9.16)	165	NA	86.5 (4-year)	92.8 (4-year)	Stage
7	Zugor	2010	Germany	NA	62	NA	NA	NA	Tumor volume, metastasis, lymph node involvement
8	Trehan	2012	India	1.6	20	NA	NA	75 (5-year)	NA
9	Yao	2012	China (Shanghai)	NA (0.33-10.58)	67	NA	78.3 (4-year)	81 (4-year)	Stage, rupture, histology
10	Guruprasad	2013	India	3.3	61	NWTS-4	83.3 (5-year)	85.2 (5-year)	Histology
11	Provenzi	2014	Brazil	5	45	NA	NA	75 (5-year)	Age at diagnosis

12[b]	Oliveira	2014	Brazil	5	50	NA	NA	75 (5-year)	Age at diagnosis
13	David	2014	UK	8.7(0.1-19.2)	97	NA	81 (5-year)	85 (5-year)	NA
14	Chan	2014	China (Hongkong)	9.2	54	NA	87 (5-year)	94 (5-year)	Metastasis
15[b]	Pan	2015	China (Shanghai)	2.6 (0.25-14.58)	142	NA	80 (5-year)	83 (5-year)	NA
16[b]	Sontes	2015	South Africa	NA	416	NA	NA	66 (5-year)	Stage
17	Njuguna	2016	Kenya	NA	39	NA	NA	41 (3-year)	Stage
18[b]	Verma	2016	India	2.5	108	NWTS-4	73 (5-year)	74 (5-year)	NA
19	John	2018	India	3	59	SIOP-2001	73 (5-year)	80 (5-year)	Metastasis
20[b]	Israels	2018	Malawi	1.33 (0.08-4.33)	73	NA	42 (5-year)	68 (5-year)	NA
21[b]	Fru	2018	Eastern Africa	NA	142	NA	NA	Zimbabwe: 33.2 Uganda:7.9 (5-year)	NA
22[b]	Seminara	2019	Argentina	NA	46	NWTS-4	70 (5-year)	87 (5-year)	Histology
23	Doganis	2019	Southern and Eastern Europe	NA	338	SIOP	85.1 (5-year)	91.1 (5-year)	Stage, histology
24	Ekenze	2019	Nigeria	1.9 (0.33-6.33)	45	NA	NA	53 (5-year)	NA

25	Bahoush	2020	Iran	4.6	52	NWTS-4	80.3 (5-year)	87 (5-year)	Sex
26	Liu	2020	Uganda	0.76 (0.15-4.2)	37	NA	NA	30 (3-year)	NA
27	Chakalaka	2020	Africa	2.25 (0.08-4.83)	201	NA	49.9 (2-year)	NA	NA
28[b]	Ghafoor	2020	Pakistan	2.35±1.91	84	SIOP-2001	75 (5-year)	78.6 (5-year)	Stage
29[b]	Nakata	2021	UK, Japan	5.3 (0.008-11.5)	1395,537	JWiTS, SIOP-2001	82.3,80.1 (5-year)	91.4,92.1 (5-year)	Sex, stage, histology
30[b]	Joaquim	2022	NA[a]	6.57	3176	SIOP-2001	85.6 (5-year)	93 (5-year)	Stage, tumor volume, histology
31	Agrawal	2022	India	1.83 (0.92-5)	156	NWTS-4	59.59 (2-year)	60.89 (2-year)	NA
32[b]	Alakaloko	2022	Nigeria	NA	40	NA	NA	75 (5-year)	Stage, histology
33	Rahiman	2022	India	2.33 (0.75-4.66)	200	SIOP-9, SIOP-2001	72 (3-year)	78.3 (3-year)	Tumor volume, malnutrition
34	Aniek	2022	Kenya	NA	92	NA	43.5 (2-year)	67.7 (2-year)	NA
35	Shyirambere	2022	Rwanda	1.5 (0.25-4.09)	136	SIOP-2001	NA	57.5 (3-year)	Tumor volume, stage
36[b]	Abdalla	2022	Sudan	5.08	143	NWTS-4	75.6 (5-year)	83.4 (5-year)	NA
37	Ekuk	2023	Uganda	1	41	NA	NA	72 (1-year)	Histology, tumor volume
38 [b]	Businge	2023	Rwanda	3.6	65	NA	NA	48.3 (5-year)	Metastasis, stage

39[b]	Zahir	2023	Iran	2 (1.00-4.17)	72	NA	NA	62 (5-year)	NA
40[b]	Koh	2023	Korea	8	342	NA	84.8 (5-year)	97.2 (5-year)	NA
41	Holmes	2023	Malawi	1.75 (1.08-2.58)	136	NA	69 (2-year)	80 (2-year)	Malnutrition, stage, vena caval tumor involvement
42	Wesevich	2023	Tanzania	2	69	NA	40 (2-year)	29 (2-year)	NA
43	Ji	2023	China (Yunnan)	NA	68	NA	NA	87.4 (5-year)	Histology
44	Nasir	2024	Nigeria	1.5 (0.25-9)	35	SIOP-2001	NA	NA	Stage
45[b]	Mergen	2024	Germany	NA	2549	SIOP 93-01, SIOP- 2001	NA	95 (5-year)	Metastasis, histology, stage
46	Mensah	2024	Africa	1.41 (0.16-2.75)	164	NA	72.6 (2-year)	NA	NA

[a]This study involves patients from 24 countries and regions, including Austria, Switzerland, Germany, Australia, Ireland, New Zealand, the United Kingdom, Denmark, Norway, Sweden, the Netherlands, Belgium, Brazil, Argentina, Croatia, the Czech Republic, Greece, Italy, Poland, Serbia, Montenegro, the Slovak Republic, Slovenia, and France.

[b]The studies were deemed suitable by two oncology research experts to represent the respective country or region in the global 5-year OS analysis and the creation of a global 5-year survival rate heatmap.
